# Efficacy of a new immunonutrition formula with extra virgin olive oil in the reduction of complications in surgeries of upper digestive tract tumors

**DOI:** 10.3389/fnut.2024.1384145

**Published:** 2024-05-28

**Authors:** Rocío Villar-Taibo, Alfonso Vidal-Casariego, Alicia Santamaría-Nieto, Ana Cantón-Blanco, Ana B. Crujeiras, Gloria Lugo Rodríguez, Gemma Rodríguez-Carnero, Francisco Pita Gutiérrez, Antía Fernández Pombo, Everardo Díaz-López, Andrea Román Eyo, Uxía Rodríguez Lavandeira, Alberto Pena-Dubra, Miguel Ángel Martínez-Olmos

**Affiliations:** ^1^Department of Endocrinology and Nutrition, Complejo Hospitalario Universitario de Santiago, Santiago de Compostela, Spain; ^2^Department of Endocrinology and Nutrition, Complejo Hospitalario Universitario de A Coruña, A Coruña, Spain; ^3^Molecular Endocrinology, Instituto de Investigación Sanitaria de Santiago (IDIS), Complejo Hospitalario Universitario de Santiago (CHUS/SERGAS), Santiago de Compostela, Spain; ^4^Epigenomics in Endocrinology and Nutrition Group, Epigenomics Unit, Instituto de Investigación Sanitaria de Santiago (IDIS), Complejo Hospitalario Universitario de Santiago (CHUS/SERGAS), Santiago de Compostela, Spain; ^5^CIBER de Fisiopatología de La Obesidad y Nutricion (CIBERobn), Instituto de Salud Carlos III, Madrid, Spain

**Keywords:** immunonutrition, arginine, omega-3 fatty acids, extra virgin olive oil, surgery, cancer

## Abstract

**Background:**

To demonstrate whether a nutritional supplement enriched with arginine, nucleotides, omega-3 fatty acids, and extra virgin olive oil reduces postoperative complications in patients with tumors in the upper digestive tract.

**Methods:**

A randomized, controlled, double-blind, multicenter clinical trial, in which a new immunomodulatory formula with extra virgin olive oil was compared with a standard isoprotein and isoenergetic formula. Patients with gastric, esophageal or biliopancreatic tumors were recruited to receive two units of immunomodulatory formula or control, 5 days before the surgical intervention.

**Results:**

A total of 119 patients were recruited. There was a significant reduction in the number of reinterventions (7.7 vs. 20.4%; *p* = 0.044) in the intervention group. There was a significant reduction in the development of fistulas in patients with phase angles >5.7°. Also, there were fewer readmissions after biliopancreatic surgeries (0.0 vs. 100%; *p* = 0.014). The length of hospital stay was similar between groups; however, with the immunomodulatory formula, the patients exhibited greater phase angle at the end of follow-up.

**Conclusion:**

The immunomodulatory formula with extra virgin olive oil administered 5 days before surgery for stomach, esophageal and biliopancreatic tumors improved cellular health and reduced postoperative complications.

**Clinical trial registration**: [https://clinicaltrials.gov/], identifier [NCT04027088].

## 1 Introduction

Malnutrition, like sarcopenia, is a risk factor for complications, infections and increased mortality after surgery for cancer of the upper digestive tract. Preoperative malnutrition in these patients is prevalent, and preoperative symptoms, including poor appetite, early satiety, and vomiting are independently associated with an increased risk of complications, morbidity, and mortality (1–4).

Surgery is a common treatment for gastric and esophageal cancer, often combined with other therapies such as chemotherapy, radiation therapy, and endoscopic techniques. Postoperative complications in upper gastrointestinal cancer surgery are common, with rates varying from 23.2 to 62%, including infectious and mechanical complications (e.g., fistulas, dehiscence), hemorrhages, as well as damage to key organs. Such as heart, lung, liver and kidney (5). The main risk factors for developing postoperative complications include comorbidities (e.g., diabetes mellitus, obesity, peripheral arterial disease, chronic renal failure), as well as an ASA classification equal to or greater than three, an operation time greater than 180 min, performance of combined organ resections, or aged over 70 years (6).

Immunonutrition (IN) is a nutritional treatment focused on: (a) modulating the inflammatory response; (b) stimulating immune function; (c) supporting intestinal trophism; and (d) reducing postoperative morbidity (7). There are several nutrients with immunomodulatory functions. Arginine is a semi-essential amino acid involved in various biological processes, such as protein synthesis, immune function, and nitric oxide production. It also plays a part in metabolism, the urea cycle, and wound healing (8, 9). Nucleotides are molecules that function as components of DNA and RNA. They play roles in cellular energy metabolism and have a part in various biochemical processes. In addition, they modulate inflammation, improve immune function (such as the action of T lymphocytes and the production of immunoglobulins) and promote the mucosal barrier function (10). Finally, omega-3 fatty acids modulate inflammation and immune function by: inhibiting lymphocyte proliferation, the production of antibodies and cytokines, the expression of adhesion molecules and the activity of natural killer cells; and triggering apoptosis (11). In patients undergoing major gastrointestinal surgeries, IN with these components could reduce complications and shorten the length of hospital stay; however, some studies have obtained discrepant results. It is worth mentioning that there is a reduced number of studies with only preoperative management, and no previous studies have used extra virgin olive oil (EVOO) as an ingredient (12).

We designed a randomized, controlled, double-blind, multicenter clinical trial in order to assess whether a new nutritional supplement with immunonutrients (arginine, nucleotides and omega-3 fatty acids) and EVOO, used preoperatively, reduced complications after oncologic surgeries of the upper digestive tract. The main goal of the present study was to assess the differences in infectious and mechanical complications, by comparing a group treated with preoperative IN and another group with an equivalent formula in caloric-protein intake; however, without immunonutrients. The secondary goal was to determine the differences between groups regarding overall postoperative mortality, length of hospital stay, analytical parameters (nutritional and immunological), body composition, and the different complications.

## 2 Materials and methods

### 2.1 Study design

This is a clinical, randomized, double-blind, parallel, multicenter study, conducted from February 2019 to September 2023, registered in “Clinical Trials” under number NCT04027088. It was designed and conducted in accordance with the Declaration of Helsinki. The study protocol, the patient information leaflet, and the informed consent form were approved by the Autonomous Research Ethics Committee of Galicia on 22nd January 2019 under number 2018/548. All patients were informed about the conditions of participation in the study and agreed to participate after signing the informed consent forms.

### 2.2 Study population

Adult patients were recruited for the present study. They were well-nourished or exhibited disease-related malnutrition. They had a diagnosis of esophageal, stomach and/or pancreatic cancer at any stage, and were going to undergo surgery after evaluation by the tumor committee. Pregnant or lactating women, patients who suffered from advanced kidney disease (glomerular filtration rate < 25 mL/min/1.73 m^2^) or had an allergy or intolerance to any of the ingredients of the formulas under study or any contraindication to the use of enteral nutrition were excluded.

### 2.3 Clinical study

The patients included in the present study were randomized into two nutritional treatment groups based on nutritional formulas of different compositions. Randomization was carried out using a numerical table generated by the Epidat 3.1 program (Consellería de Sanidade, Xunta de Galicia, Spain; Pan American Health Organization (PAHO-WHO); University CES, Colombia.), following a 1:1 ratio. Each patient received a participant number that assigned them to a specific group and receive one nutritional formula or another. The distribution between groups followed a 1:1 ratio.

Two visits were performed, namely, V1 (initial, 1 week before surgery) and V2 (final, the day before surgery). Demographic data (sex and age) and clinical data (related to oncological diagnosis, antineoplastic treatment, and surgical treatment) were collected, and anthropometric and body composition measurements were performed in the two visits. In order to know the impact of nutritional supplementation at a clinical-surgical level, the data, collected from the medical records, were those related to: surgical complications that occurred during hospital admission (post-surgical fistulas, dehiscence, intra-abdominal abscesses, surgical wound infections, pancreatitis, extra-abdominal infections, deaths); surgical reinterventions; length of hospital stay; rate of hospital admissions; and emergency room attendances, during the 30 days after hospital discharge.

### 2.4 Nutritional treatment

The patients received two daily containers of the treatment under study or the control treatment during the 5 days prior to the surgical interventions.

Formulas under study (Table 1):

- Immunomodulator: Bi1 Procare^®^ (Adventia Pharma): High-calorie and high-protein oral nutritional supplement, with fiber, EVOO, and the following immunonutrients: arginine; omega-3 fatty acids (eicosapentaenoic acid [EPA] and docosahexaenoic acid [DHA]); and nucleotides.
- Standard: Bi1 1.5 HP^®^ (Adventia Pharma): standard polymeric high-calorie and high-protein oral nutritional supplement, without fiber, EVOO or immunonutrients.

**Table 1:** Composition of macronutrients and ingredients of the formulas per 100 mL.

	Immunonutrition (a)	Control (b)
Energy (kcal)	150	159
Proteins (g) (TE%) Ingredients Arginine (g)	8 (21.4%) Caseinate (31.6%), whey protein concentrate (45%) and L-arginine (23.4%) 2.01	7.88 (21%) Caseinate (72%) and whey protein concentrate (28%) 0.26
Carbohydrates (g) (TE%) Ingredients Sugars	15.1 (40.3%) Dextrin (55%) and maltodextrin (45%) 1.18	18 (48%) Maltodextrin (100%) 3.1
Fat (g) (TE%) Ingredients EPA (mg) DHA (mg)	5.9 /35.6% EVOO (40%), MCT (30%), fish oil (17%), and canola oil (13%) 462 288	5.2 (31%) Canola oil (26%) and high oleic sunflower oil (74%) 0 0
Fibre (g) Soluble/insoluble Ingredients	2.05 100/0 Fructooligosaccharides	0 – –
Nucleotides (mg) Ingredients	200 RNA	0 –

DHA, Docosahexaenoic acid; EPA, Eicosapentaenoic acid; EVOO, Extra virgin olive oil; MCT, Medium chain triglyceride; RNA, Ribonucleic acid; TE%, Percentage of total energy; (a), Bi1 procare^®^, Adventia Pharma S.L, Spain; (b), Bi1 1.5 hp^®^, Adventia Pharma S.L., Spain.

The nutritional formulas were provided in unlabeled Tetra Pak^®^ containers (200 mL) that were exactly the same for both formulas. Each container had a numerical code as the only differentiation between both products. This way, the patients received the experimental supplement (IN) or the control formula. An assessment of the daily intake of nutritional supplements was performed using written records self-completed by the patients in order to determine adherence to the nutritional treatments (13).

### 2.5 Anthropometric study

Body weight and height were measured using a calibrated stadiometer and a scale with the patients wearing light clothing and no shoes. Body mass index (BMI) was calculated according to current weight and the percentage of weight lost from usual weight.

### 2.6 Body composition

Body composition was assessed using a single-frequency bioimpedance technique (50 kHz) obtaining the resistance, reactance, and phase angle values. Based on the aforementioned values, the appendicular skeletal muscle index, fat mass, and lean mass were obtained using the AKERN© measuring device (Akern S.L., Pisa, Italy). Bioimpedance was performed with the subject in a supine position on a non-conductive surface, with the limbs abducted at 45°. Fasting for more than 2 h was recommended, avoiding the intake of alcohol, coffee, caffeinated soft drinks, and chocolates in the previous 24 h, as well as vigorous exercise.

### 2.7 Functional status

Hand grip strength was measured using dynamometry. To that end, a JAMAR HAND^®^ dynamometer was used taking measurements three times in each hand alternately and obtaining the average of these results. The diagnosis of malnutrition was made following the criteria of the Global Clinical Nutrition Community.

### 2.8 Biochemical analysis

Determinations of prealbumin, total cholesterol, CD4 and CD8 T lymphocytes, retinol-binding protein, and C-reactive protein were performed.

### 2.9 Statistical analysis

The calculation for the sample size was performed according to the results of Klek’s meta-analysis (14). Establishing a significant difference in complications (14%), with 95% confidence interval and a power of 80%, the number of patients obtained was 178, considering potential losses of 10%.

Categorical data are presented as percentages, and quantitative data as means (standard deviation). Normality of continuous quantitative data was confirmed using the Kolmogorov–Smirnov test. Categorical variables were compared using the chi-square test. Quantitative variables were compared with the Student’s *t*-test for independent samples (comparison between groups) and for related samples (comparison between visits). The correlations between continuous quantitative variables were assessed using Pearson test. The assessment of the frequency of complications was performed according to different subgroups, namely: sex; mean age; nutritional status; tumor diagnosis; previous neoadjuvant treatment; or mean phase angle. A multivariate analysis with linear regression was performed when it was necessary to adjust a variable. A *p*-value <0.05 was considered significant. The analysis of the results was performed using the BM SPSS Statistics 21 software.

## 3 Results

### 3.1 Characteristics of the patients

**Figure 1:**
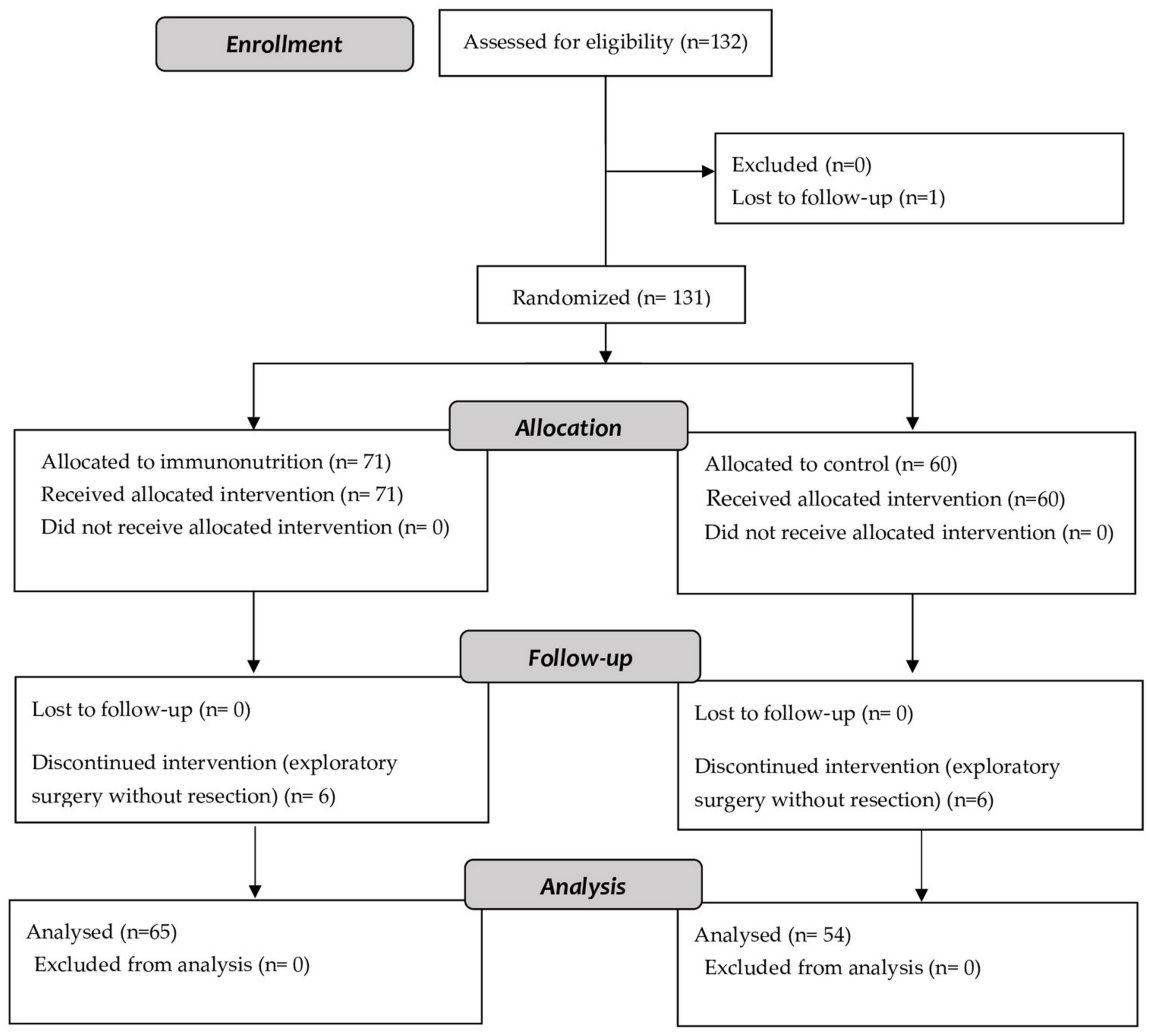
Flowchart.

One hundred thirty-two patients were recruited for the present study. Of these patients, 65 finally received the immunomodulatory formula and 54 the control formula (Figure 1).

One hundred eleven patients (93.3%) completed the protocol, with no differences between the groups in the number of dropouts (IN 95.4% vs. control 90.7%; *p* = 0.314). The main characteristics of the patients are summarized in Table 2. Before the intervention, 56.6% of the patients in the intervention group received neoadjuvant therapy, and 43.8% in the control group (*p* = 0.251). The prevalence of malnutrition was 48.4% in patients who received IN, and 53.7% in those who received the control formula (*p* = 0.569). There were no differences between groups in the prevalence of malnutrition related to the stage 2 disease (21.9% vs. 27.8%; *p* = 0.746).

**Table 2:** Baseline characteristics of patients according to groups.

	Immunonutrition (*n* = 65)	Control (*n* = 54)	*p*-value
Age (years)	68.2 (10.7)	70.0 (9.6)	0.348
Men (%)	70.8	75.9	0.528
Tobacco use (%)	13.8	13.0	0.732
Diabetes (%)	35.4	41.5	0.496
AHT (%)	56.9	55.6	0.881
Dyslipidemia (%)	36.9	42.6	0.529
CKD (%)	10.8	3.7	0.147
Weight (kg)	72.7 (15.5)	73.3 (14.8)	0.820
BMI (kg/m^2^)	26.5 (4.6)	27.1 (4.9)	0.527
Weight loss (%)	6.9 (9.1)	8.1 (9.2)	0.467
Prealbumin (mg/dL)	25.1 (6.4)	23.7 (5.4)	0.259
CRP (mg/dL)	0.8 (1.3)	0.7 (1.0)	0.523
Cholesterol (mg/dL)	175.0 (46.9)	171.7 (42.2)	0.718
Lymphocytes (10^9^/L)	1.7 (1.4)	1.5 (0.7)	0.292
Diagnostic (%)			
Gastric neoplasia	61.5 (40)	74.1 (40)	0.346
Esophageal neoplasia	32.3 (21)	22.2 (12)	
Biliopancreatic neoplasia	6.2 (4)	3.7 (2)	

AHT, Arterial hypertension; CKD, Chronic kidney disease; BMI, Body mass index; CRP, C-reactive protein.Quantitative data are presented as means (standard deviation).

### 3.2 Postsurgical complications

Of the 119 patients recruited, 59 exhibited some complication in the postoperative period, the most frequent being extra-abdominal infections (21%), dehiscence (7.6%), fistulas (4.2%), surgical wound infections (4.2%), intra-abdominal abscesses (3.2%), and pancreatitis (0.8%). In addition, 13.4% required being reintervened, 5% were readmitted within 30 days, 6.8% went to the emergency room, and only 2.5% died. The average length of hospital stay was 17.7 (19.3) days.

**Figure 2:**
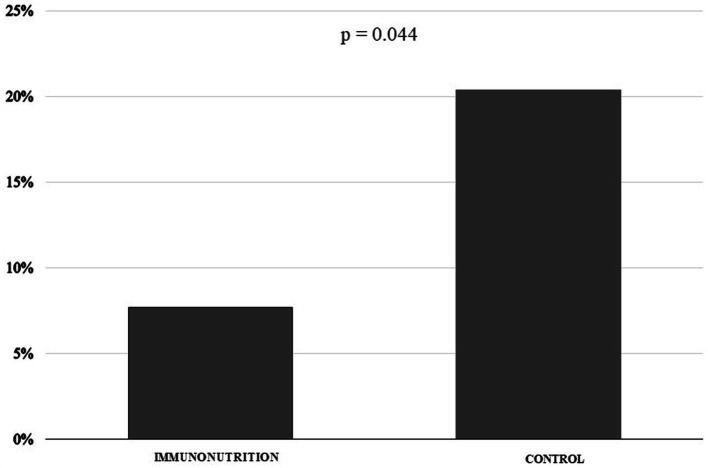
Frequency of reinterventions.

When the differences between treatment groups were assessed (Table 3), a trend toward a lower prevalence of extra-abdominal infections, dehiscence, fistulas, intra-abdominal infections, emergency care, and deaths was observed in the intervention group in comparison to the control group; although these differences did not reach statistical significance. With respect to the length of hospital stay, no statistically significant differences were observed between groups (*p* = 0.716). However, the intervention group stayed a lower number of days hospitalized (16.9 [16.9] days) in comparison to the control group (18.6 [21.9] days). It is worth mentioning that a significant reduction was observed in the number of reinterventions in the intervention group (IN 7.7% vs. control 20.4%; *p* = 0.044) (Figure 2).

**Table 3:** Frequency of complications after surgery according to the treatment group.

	Immunonutrition (*n* = 65)	Control (*n* = 54)	*p*-value
Extra abdominal infections % (n)	20.0 (13)	22.2 (12)	0.767
Dehiscence % (n)	6.2 (4)	9.3 (5)	0.524
Fistulas % (n)	3.1 (2)	5.6 (3)	0.502
Surgical wound infections % (n)	3.1 (2)	5.6 (3)	0.502
Intra-abdominal abscesses % (n)	3.1 (2)	3.7 (2)	0.850
Pancreatitis % (n)	0.8 (1)	0.0 (0)	0.360
Readmissions % (n)	3.1 (2)	7.4 (4)	0.282
Emergency care % (n)	4.6 (3)	9.4 (5)	0.300
Deaths % (n)	1.5 (1)	3.7 (2)	0.453
Total infectious complications % (n)	26.2 (17)	25.9 (14)	0.978
Total mechanical complications % (n)	9.2 (6)	14.8 (8)	0.347

Data are presented as percentage (%) and absolute frequency (n).

In the analysis by subgroup, the use of IN was associated with a lower frequency of reinterventions in men and in those patients with gastric and esophageal tumors, whereas in well-nourished patients, the result was on the limit of statistical significance. In patients with a phase angle above the mean, there was a significant reduction in the development of fistulas with the immuno-modulatory formula (Table 4). There were no differences between subgroups in terms of readmissions, emergency room visits or deaths (data not shown).

**Table 4:** Differences in the prevalence of complications between the subgroups age, sex, nutritional status, tumor diagnosis and neoadjuvant therapy.

	Immunonutrition (*n* = 65)	Control (*n* = 54)	*p*-value
Extra-abdominal infections			
Age			
<69 years	24.2% (8)	31.6% (6)	0.566
≥69 years	15.6% (5)	17.1% (6)	0.867
Sex			
Men	23.9% (11)	24.4% (10)	0.959
Women	10.5% (2)	15.4% (2)	0.683
Nutritional status			
Normal	21.2% (7)	20.0% (5)	0.910
DRM	19.4% (6)	24.1% (7)	0.653
Tumor diagnosis			
Gastric	12.5% (5)	17.5% (7)	0.531
Esophageal	38.1% (8)	41.7% (5)	0.840
Biliopancreatic	0.0% (0)	0.0% (0)	-
Neoadjuvant			
Yes	20.0% (6)	21.4% (3)	0.913
No	8.7% (2)	16.7% (3)	0.439
Phase angle			
<5.7°	16.7% (4)	26.9% (7)	0.382
≥5.7°	20.6% (7)	20.0% (4)	0.959
Dehiscence			
Age			
<69 years	6.1% (2)	5.3 (1)	0.905
≥69 years	6.3% (2)	11.4% (4)	0.458
Sex			
Men	6.5% (3)	7.3% (3)	0.884
Women	5.3% (1)	15.4% (2)	0.335
Nutritional condition			
Normal	6.1% (2)	12.0% (3)	0.425
DRM	6.5% (2)	6.9% (2)	0.945
Tumor diagnosis			
Gastric	2.5% (1)	7.5% (3)	0.305
Esophageal	14.3% (3)	16.7% (2)	0.854
Biliopancreatic	0.0% (0)	0.0% (0)	-
Neoadjuvant therapy			
Yes	3.3% (1)	0.0% (0)	0.490
No	4.3% (1)	5.6% (1)	0.859
Phase angle			
<5.7°	8.3% (2)269	15.4% (4)	0.443
≥5.7°	5.9% (2)	0.0% (0)	0.269
Fistulas			
Age			
<69 years	0.0% (0)	5.3% (1)	0.183
≥69 years	6.3% (2)	5.7% (2)	0.926
Sex			
Men	2.2% (1)	7.3% (3)	0.253
Women	5.3% (1)	0.0% (0)	0.401
Nutritional status			
Normal	3.0% (1)	4.0% (1)	0.841
DRM	3.2% (1)	6.9% (2)	0.514
Tumor diagnosis			
Gastric	2.5% (1)	5.0% (2)	0.556
Esophageal	4.8% (1)	8.3% (1)	0.679
Biliopancreatic	0.0% (0)	0.0% (0)	–
Neoadjuvant therapy			
Yes	3.3% (1)	0.0% (0)	0.490
No	0.0% (0)	11.1% (2)	0.101
Phase angle			
<5.7°	4.2% (1)	0.0% (0)	0.293
≥5.7°	0.0% (0)	15.0% (3)	0.020
Surgical wound infections			
Age			
<69 years	3.0% (1)	10.5% (2)	0.264
≥69 years	3.1% (1)	2.9% (1)	0.949
Sex			
Men	4.3% (2)	4.9% (2)	0.906
Women	0.0% (0)	7.7% (1)	0.219
Nutritional status			
Normal	3.0% (1)	8.0% (2)	0.397
DRM	3.2% (1)	3.4% (1)	0.962
Tumor diagnosis			
Gastric	2.5% (1)	5.0% (2)	0.556
Esophageal	4.8% (1)	8.3% (1)	0.679
Biliopancreatic	0.0% (0)	0.0% (0)	–
Neoadjuvant therapy			
Yes	3.3% (1)	7.1% (1)	0.572
No	4.3% (1)	5.6% (1)	0.859
Phase angle			
< 5.7°	4.2% (1)	3.8% (1)	0.954
≥ 5.7°	2.9% (1)	10.0% (2)	0.274
Intra-abdominal abscesses			
Age			
<69 years	3.0% (1)	0.0% (0)	0.444
≥69 years	3.1% (1)	5.7% (2)	0.609
Sex			
Men	0.0% (0)	4.9% (2)	0.130
Women	10.5% (2)	0.0% (0)	0.227
Nutritional status			
Normal	0.0% (0)	4.0% (1)	0.246
DRM	6.5% (2)	3.4% (1)	0.594
Tumor diagnosis			
Gastric	2.5% (1)	2.5% (1)	1.000
Esophageal	0.0% (0)	8.3% (1)	0.179
Biliopancreatic	25.0% (1)	0.0% (0)	0.439
Neoadjuvant therapy			
Yes	0.0% (0)	0.0% (0)	-
No	4.3% (1)	5.6% (1)	0.859
Phase angle			
<5.7°	4.2% (1)	3.8% (1)	0.954
≥5.7°	0.0% (0)	5.0% (1)	0.188
Reinterventions			
Age			
<69 years	6.1% (2)	21.1% (4)	0.103
≥69 years	9.4% (3)	20.0% (7)	0.223
Sex			
Men	6.5% (3)	24.4% (10)	0.020
Women	10.5% (2)	7.7% (1)	0.787
Nutritional condition			
Normal	6.1% (2)	24.0% (6)	0.048
DRM	9.7% (3)	17.2% (5)	0.389
Tumor diagnosis			
Gastric	2.5% (1)	15.0% (6)	0.048
Esophageal	9.5% (2)	41.7% (5)	0.030
Biliopancreatic	50.0% (2)	0.0% (0)	0.221
Neoadjuvant therapy			
Yes	3.3% (1)	14.3% (2)	0.179
No	4.3% (1)	16.7% (3)	0.187
Phase angle			
<5.7°	4.2% (1)	19.2% (5)	0.101
≥5.7°	5.9% (2)	20.0% (4)	0.111

DRM, Disease-related malnutrition. Data are presented as percentage (%) and absolute frequency (n).

### 3.3 Evolution of anthropometric parameters and body composition

**Figure 3:**
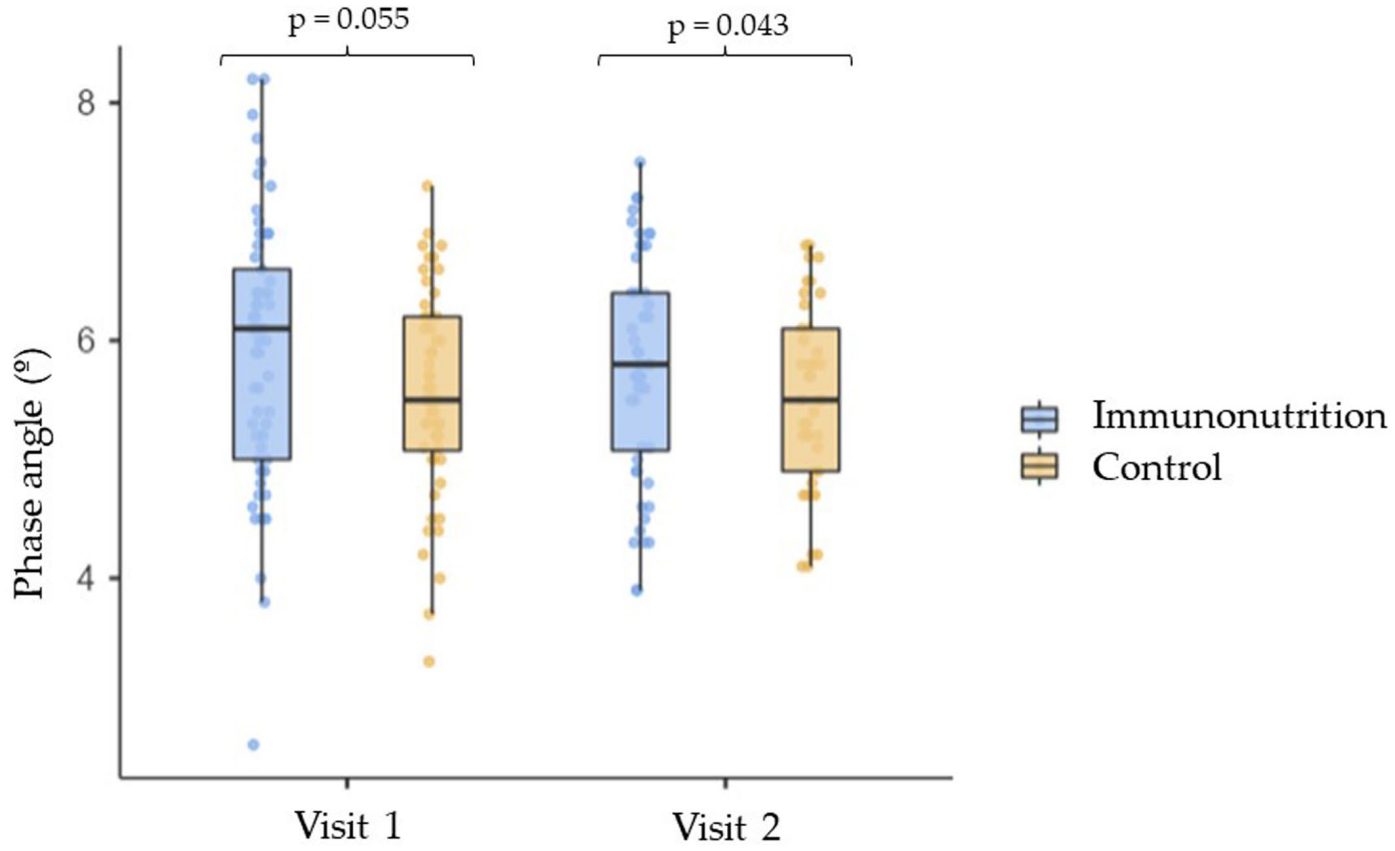
Evaluation of the phase angle.

Between the two study visits, there were significant reductions in weight, BMI and dynamometry in both groups, with no statistically significant differences between the intervention group and the control group. However, there were no changes in fat mass, BMI and appendicular skeletal muscle index (Table 5). A relevant result was observed in the phase angle after the nutritional intervention. Patients who received IN had greater phase angles than those in the control group (5.9 [0.9]° vs. 5.4 [0.8] °; *p* = 0.043) (Figure 3).

**Table 5:** Evolution of anthropometry and body composition.

	Immunonutrition (*n* = 65)		Control (*n* = 54)		*p*-value			
	Initial visit	Final visit	Initial visit	Final visit	Between groups—V1	Between groups—V2	Immunonutrition V1-V2	Control V1-V2
Weight (kg)	72.7 (15.5)	70.4 (13.2)	73.3 (14.8)	69.5 (14.2)	0.820	0.776	<0.001	0.001
BMI (kg/m^2^)	26.5 (4.6)	26.2 (4.2)	27.1 (4.9)	26.0 (4.9)	0.527	0.858	<0.001	0.001
ASMI (kg/m^2^)	7.8 (1.3)	7.9 (2.0)	7.8 (1.4)	7.5 (1.1)	0.913	0.224	0.868	0.157
LMI (kg/m^2^)	21.3 (3.1)	20.4 (3.9)	21.0 (3.1)	19.9 (3.1)	0.625	0.546	0.039	0.146
Fat mass (%)	20.3 (9.1)	19.6 (8.0)	21.5 (9.9)	22.7 (15.6)	0.531	0.247	0.599	0.294
Dynamometry (kg)	29.8 (11.0)	29.1 (11.7)	28.8 (10.0)	25.4 (8.8)	0.632	0.114	<0.001	<0.001

V1, Initial visit; V2, Final visit; BMI, Body mass index; ASMI, Appendicular skeletal muscle index; LMI, Lean mass index.

### 3.4 Evolution of analytical parameters

There were significant reductions in the levels of prealbumin, total cholesterol, and CD4 and CD8 T lymphocytes in the two groups of the study. On the other hand, retinol-binding protein levels were only reduced in the control group (Table 6). The CD4/CD8 ratio was significantly higher in the two visits in the group that received IN. After adjusting for the type of formula received and the initial ratio, no significant effect of treatment was observed on the final CD4/CD8 ratio (*β* = 1.02 [95% CI −101.3 to 103.3]; *p* = 0.984).

**Table 6:** Evolution of biochemical and immunological parameters.

	Immunonutrition (*n* = 65)		Control (*n* = 54)		*p*-value			
	Initial visit	Final visit	Initial visit	Final visit	Between groups—V1	Between groups—V2	Immunonutrition V1-V2	Control V1-V2
Prealbumin (mg/dL)	25.1 (6.4)	20.2 (4.3)	23.7 (5.4)	19.7 (5.9)	0.259	0.663	<0.001	<0.001
RBP (mg/dL)	5.7 (5.4)	4.2 (1.0)	4.7 (1.6)	4.0 (1.3)	0.324	0.489	0.102	0.009
Cholesterol (mg/dL)	175.0 (46.9)	172.0 (65.6)	171.7 (42.2)	153.7 (40.4)	0.718	0.189	0.005	0.002
Lymphocytes (10^9^/L)	1.71 (1.4)	1.35 (0.9)	1.47 (0.7)	2.13 (5.9)	0.292	0.336	0.214	0.430
CD4 (Cell/μL)	714.5 (343.8)	549.5 (375.7)	650.5 (333.1)	508.4 (311.6)	0.359	0.573	<0.001	<0.001
CD8 (Cell/μL)	405.9 (397.1)	315.3 (299.9)	439.2 (278.3)	352.2 (236.8)	0.642	0.520	0.003	0.006
CD4/CD8 ratio	2.91 (2.65)	2.70 (2.66)	1.84 (1.18)	1.83 (1.18)	0.010	0.038	0.960	0.694
CRP (mg/dL)	0.8 (1.3)	0.6 (0.6)	0.7 (1.0)	0.8 (1.3)	0.523	0.260	0.194	0.856

V1, Initial visit; V2, Final visit; RBP, Retinol-binding protein; CRP, C-reactive protein.

At the end of follow-up, a statistically significant correlation between phase angle, BMI, appendicular skeletal muscle index, lean mass index and dynamometry was evident in all patients. Analyzing by subgroups, the patients who had received the immunomodulatory formula presented a statistically significant correlation between this parameter and the number of CD4 and CD8 T lymphocytes, which was not found in the control group (Table 7).

**Table 7:** Correlation between the final phase angle and body composition and analytical parameters.

	Immunonutrition (*n* = 65)			Control (*n* = 54)			Total		
	*r*	95% CI	*p*-value	*r*	95% CI	*p*-value	*r*	95% CI	*p*-value
Weight (kg)	0.118	−0.172 to 0.389	0.423	0.223	−0.136 to 0.531	0.219	0.161	−0.060 to 0.368	0.153
BMI (kg/m^2^)	0.288	0.004 to 0.529	0.047	0.187	−0.173 to 0.503	0.305	0.836	0.756 to 0.891	<0.001
IMEA (kg/m^2^)	0.162	−0.125 to 0.424	0.267	0.437	0.132 to 0.667	0.007	0.475	0.285 to 0.269	<0.001
LMI (kg/m^2^)	0.348	0.071 to 0.576	0.015	0.357	0.037 to 0.610	0.030	0.467	0.274 to 0.624	<0.001
Fat mass (%)	−0.105	−0.381 to 0.187	0.480	−0.111	−0.442 to 0.248	0.547	0.193	−0.029 to 0.397	0.088
Dynamometry (kg)	−0.090	−0.362 to 0.196	0.538	0.300	−0.032 to 0.572	0.076	0.445	0.252 to 0.604	<0.001
Prealbumin (mg/dL)	0.214	−0.081 to 0.475	0.153	0.165	−0.183 to 0.76	0.351	0.133	−0.092 to 0.345	0.245
RBP (mg/dL)	−0.002	−0.306 to 0.302	0.989	−0.076	−0.450 to 0.321	0.714	0.215	−0.021 to 0.428	0.074
Lymphocytes (10^9^/L)	0.345	0.057 to 0.580	0.020	−0.047	−0.384 to 0.301	0.795	−0.097	−0.315 to 0.132	0.407
CD4 (Cell/μL)	0.341	0.049 to 0.579	0.023	−0.163	−0.480 to 0.191	0.363	0.141	−0.088 to 0.357	0.226
CD8 (Cell/μL)	0.362	0.074 to 0.595	0.016	−0.221	−0.525 to 0.132	0.216	0.108	−0.122 to 0.327	0.358
CD4/CD8 ratio	−0.078	−0.367 to 0.224	0.614	0.093	−0.258 to 0.423	0.606	0.022	−0.206 to 0.248	0.851
CRP (mg/dL)	−0.043	−0.335 to 0.257	0.782	−0.192	−0.527 to 0.195	0.328	−0.022	−0.213 to 0.254	0.858

BMI, Body mass index; ASMI, Appendicular skeletal muscle index; LMI, Lean mass index; RBP, Retinol-binding protein; CRP, C-reactive protein.

## 4 Discussion

In the present study, the administration of an immunomodulatory formula with EVOO in patients with upper digestive tumors, 5 days before surgery, was associated with a lower frequency of surgical reinterventions. In some groups of patients, it was associated with reduced development of fistulas, and fewer readmissions. Phase angle improved with IN, and this improvement was correlated with immunological and body composition parameters.

On an overall basis, the use of IN produced benefits reducing postsurgical complications. Thus, in a meta-analysis of 27 clinical trials, it was found that IN reduced infectious complications. The reduction in these complications was greater in the perioperative regimen (54%) than in the preoperative (42%) or postoperative (37%) regimens. Furthermore, perioperative and postoperative IN were associated with shorter length of hospital stay, and only perioperative IN was able to reduce non-infectious complications (15). Another meta-analysis of 61 randomized trials conducted with patients undergoing surgery for different upper digestive and head and neck tumors found that perioperative IN promoted a reduction in infectious complications and length of hospital stay, without a decrease in mortality (16). In the meta-analyses conducted by Cerantola et al. and Niu et al., perioperative IN reduced the occurrence of total and infectious complications, as well as the length of hospital stay. On the other hand, perioperative IN further reduced total complications, and postoperative IN further reduced infectious complications (17, 18).

Although the perioperative administration of IN seems to be the most beneficial regimen to reduce complications in surgeries for digestive tract cancer, we decided to perform an exclusive preoperative approach with IN administered 5 days before the surgical interventions. A previous meta-analysis had indicated that a minimum of 5 days of IN was capable of reducing infectious complications by 48% and length of hospital stay by 1.5 days (19). Regarding the time of nutritional treatment, it was chosen to administer it preoperatively with the goal that the patients came to surgery with a better nutritional and immune status; however, it was not continued in the postoperative period because patient management was more heterogeneous between centers.

When the focus was determining the type of tumor in which IN would be most effective, the study conducted by Shen found the best results in colorectal surgery, although benefits have also been found in other gastric and pancreatic tumors (17, 20, 21). With respect to esophageal surgery, there are slightly more heterogeneous results (22–24). In the present study, the improvement in the rate of surgical reinterventions was consistent in the two major groups of tumors included, i.e., gastric and esophageal, supporting the evidence of the benefit caused by IN in both groups of patients.

In our study, no significant changes were observed in the levels of CD4 and CD8 T lymphocytes after the intervention. This result is different to the results obtained in previous studies. Surgical aggression produces reductions in the populations of these lymphocytes and increases in the expression of pro-inflammatory cytokines. Most studies have indicated better recovery of cellular immunity and attenuation of the inflammatory response with the pre-or perioperative administration of an immunomodulatory formula. However, in the present study, the evolution of these parameters after the interventions was not assessed (25, 26). The CD4/CD8 ratio, an indirect marker of immune function, was similar in both groups both before and after the nutritional intervention. This ratio is considered decreased when it is less than 1.38. In our study, the levels found were above that value, which could indicate that the recruited patients did not exhibit significant immunosuppression and, therefore, explain why a significant effect on complications was not observed (27). The formulas have different content of fiber. Dietary fiber could have an immunomodulatory effect through several mechanisms, including the modulation of gut microflora, production of short chain fatty acids, and direct interactions with immune cells. These effects can influence both innate and adaptive immune responses and may vary depending on the type of fiber and its source. The studied formula contained fructooligosaccharides (FOS), which supplementation can enhance immune function by increasing immunoglobulin A concentrations in gut and serum. Nevertheless, there were no differences in the infection rates between groups (28).

A relevant fact of the present study is that the use of IN was associated with greater phase angle at the end of follow-up, and only in patients who had received IN. This bioelectric parameter was positively correlated with the number of total lymphocytes and subpopulations of CD4 and CD8. As a gross variable derived from bioelectrical impedance analysis, phase angle reflects the relationship between resistance and reactance, and is, therefore, an indicator of the health and integrity of the cell membrane. Higher values reflect better cellular function, greater muscle mass and lower fat mass, as well as better distribution of body water. Phase angle is an independent predictor of deterioration in nutritional and functional status and survival (29, 30). There is evidence that the phase angle is negatively correlated with proinflammatory molecules such as CRP, TNF-α, interleukin-6 and interleukin-10, both in healthy people and in chronic disease situations. Some data also point to the relationship between the phase angle and markers of oxidative stress and antioxidant intake (31, 32). In patients undergoing surgery for head and neck cancer, the use for 8 days of an immunomodulatory formula, with a composition similar to that used in the present study, produced a significant increase in the phase angle (33). The results obtained in the present study indicated a relationship between phase angle and immune function, and the potential usefulness to detect early changes in inflammation.

Among the strengths of the study, it is worth highlighting its robust design in the form of a double-blind clinical trial, in which a control formula was used with the same calorie and protein intake as the study formula. This design allowed us to differentiate the effects of immunonutrients from the nutritional contribution of a standard formula. For the diagnosis of malnutrition, the Global Leadership Initiative on Malnutrition criteria were used, when previous studies used more non-specific malnutrition criteria such as percentage of weight loss, BMI or hypoalbuminemia. Furthermore, the use of single-frequency bioimpedance to estimate body composition and be able to assess parameters such as phase angle has been uncommon in studies conducted with IN.

Regarding the weaknesses of the study, it should be noted that the planned sample size was not reached due to the COVID-19 epidemic, which reduced the power of the study. This fact, together with the low frequency of complications, has limited the possibility of finding more statistically significant results. The five-day intervention proposed in the present study was satisfactory to find clinical and phase angle benefits. It is possible to hypothesize whether a nutritional intervention performed for a longer period of time or that includes the postoperative period could result in better clinical, immunological or body composition results.

## 5 Conclusion

The use of a new immunomodulatory formula enriched with arginine, nucleotides, n-3 fatty acids, and whose main source of fat was EVOO, for 5 days before surgical treatment of gastric, esophageal or biliopancreatic tumors significantly reduced the number of reinterventions. The benefits of the immunomodulatory formula can be also expected in patients with a good nutritional status.

The phase angle improved with IN and could be related to an improvement in cellular health and immune function. The results of the present study reinforce the role of IN in modifying the prognosis of patients treated with oncological surgeries, even in a short period of nutritional intervention before surgeries. The potential benefits of a longer preoperative intervention or one that includes the postoperative period could be the subject of future studies.

## Data availability statement

The raw data supporting the conclusions of this article will be made available by the authors, without undue reservation.

## Ethics statement

The studies involving humans were approved by the Autonomous Research Ethics Committee of Galicia. The studies were conducted in accordance with the local legislation and institutional requirements. The participants provided their written informed consent to participate in this study.

## Author contributions

RV-T: Conceptualization, Investigation, Writing – original draft. AV-C: Conceptualization, Investigation, Methodology, Writing – original draft. AS-N: Data curation, Writing – original draft. AC-B: Investigation, Writing – review & editing. ABC: Conceptualization, Formal analysis, Methodology, Supervision, Writing – review & editing. GLR: Investigation, Writing – review & editing. GR-C: Data curation, Investigation, Writing – review & editing. FPG: Conceptualization, Investigation, Writing – review & editing. AFP: Investigation, Writing – review & editing. ED-L: Data curation, Investigation, Writing – review & editing. ARE: Investigation, Writing – review & editing. URL: Data curation, Investigation, Writing – review & editing. AP-D: Data curation, Investigation, Writing – review & editing. MM-O: Conceptualization, Supervision, Writing – review & editing.

## References

[1] Doyle S Mongan A Donohoe C Pidgeon G Sherlock M Reynolds J. Impact of visceral obesity and metabolic syndrome on the postoperative immune, inflammatory, and endocrine response following surgery for esophageal adenocarcinoma. Dis Esophagus. (2017) 30:1–11. doi: 10.1093/dote/dox008, PMID: 28475745

[2] Deftereos I Yeung JMC Arslan J Carter VM Isenring E Kiss N. Assessment of nutritional status and nutrition impact symptoms in patients undergoing resection for upper gastrointestinal cancer: results from the multi- Centre NOURISH point prevalence study. Nutrients. (2021) 13:3349. doi: 10.3390/nu13103349 34684353 PMC8539371

[3] Kakavas S Karayiannis D Bouloubasi Z Poulia P Kompogiorgas S Konstantinou D. Global leadership initiative on malnutrition criteria predict pulmonary complications and 90-day mortality after major abdominal surgery in cancer patients. Nutrients. (2020) 12:3726. doi: 10.3390/nu12123726, PMID: 33287107 PMC7761640

[4] Garth A Newsome C Simmance N Crowe T. Nutritional status, nutrition practices and post-operative complications in patients with gastrointestinal cancer. J Hum Nutr Diet. (2010) 23:393–401. doi: 10.1111/j.1365-277X.2010.01058.x, PMID: 20337847

[5] Szakmany T Ditai J Kirov M Protsenko D Osinaike B Venara A. In-hospital clinical outcomes after upper gastrointestinal surgery: data from an international observational study. Eur J Surg Oncol. (2017) 43:2324–32. doi: 10.1016/j.ejso.2017.08.002 28916417

[6] Xu Y Wang Y Xi C Ye N Xu X. Is it safe to perform gastrectomy in gastric cancer patients aged 80 or older? A meta-analysis and systematic review. Medicine. (2019) 98:e16092. doi: 10.1097/MD.0000000000016092, PMID: 31192972 PMC6587649

[7] García-Malpartida K Aragón-Valera C Botella-Romero F Ocón-Bretón MJ López-Gómez JJ. Effects of immunonutrition on Cancer patients undergoing surgery: a scoping review. Nutrients. (2023) 15:1776. doi: 10.3390/nu15071776, PMID: 37049616 PMC10096769

[8] Jin-Ming W Lin M-T. Effects of specific nutrients on immune modulation in patients with gastrectomy. Ann Gastroenterol Surg. (2019) 4:14–20. doi: 10.1002/ags3.12299 32021954 PMC6992678

[9] Efron D Barbul A. Role of arginine in immunonutrition. J Gastroenterol. (2000) 35:20–3. 10779211

[10] Grimble R. Nutritional modulation of immune function. Proc Nutr Soc. (2001) 60:389–97. doi: 10.1079/PNS2001102 11681814

[11] Martínez VC. Nuevos nutrientes en nutrición enteral. Nutr Hosp. (2000) 15:69–74. 11220005

[12] Marimuthu K Varadhan K Ljungqvist O Lobo D. A meta-analysis of the effect of combinations of immune modulating nutrients on outcome in patients undergoing major open gastrointestinal surgery. Ann Surg. (2012) 255:1060–8. doi: 10.1097/SLA.0b013e318252edf8, PMID: 22549749

[13] Seguy D Hubert H Robert J Meunier JP Guérin O Raynaud-Simon A. Compliance to oral nutritional supplementation decreases the risk of hospitalization in malnourished older adults without extra health care cost: prospective observational cohort study. Clin Nutr. (2020) 39:1900–7. doi: 10.1016/j.clnu.2019.08.005, PMID: 31471163

[14] Klek S Szybinski P Szczepanek K. Perioperative immunonutrition in surgical cancer patients: a summary of a decade of research. World J Surg. (2014) 38:803–12. doi: 10.1007/s00268-013-2323-z, PMID: 24178185 PMC3956976

[15] Song GM Tian X Zhang L Ou YX Yi LJ Shuai T. Immunonutrition support for patients undergoing surgery for gastrointestinal malignancy: preoperative, postoperative, or perioperative? A Bayesian network Meta-analysis of randomized controlled trials. Medicine. (2015) 94:e1225. doi: 10.1097/MD.0000000000001225, PMID: 26200648 PMC4602990

[16] Yu K Zheng X Wang G Liu M Li Y Yu P. Immunonutrition vs standard nutrition for Cancer patients: a systematic review and Meta-analysis (part 1). J Parenter Enter Nutr. (2020) 44:742–67. doi: 10.1002/jpen.1736, PMID: 31709584

[17] Cerantola Y Hübner M Grass F Demartines N Schäfer M. Immunonutrition in gastrointestinal surgery. Br J Surg. (2011) 98:37–48. doi: 10.1002/bjs.7273, PMID: 20931620

[18] Niu JW Zhou L Liu ZZ Pei DP Fan WQ Ning W. A systematic review and Meta-analysis of the effects of perioperative immunonutrition in gastrointestinal Cancer patients. Nutr Cancer. (2021) 73:252–61. doi: 10.1080/01635581.2020.1749291, PMID: 32285694

[19] Adiamah A Skořepa P Weimann A Lobo DN. The impact of preoperative immune modulating nutrition on outcomes in patients undergoing surgery for gastrointestinal cancer: a systematic review and meta-analysis. Ann Surg. (2019) 270:247–56. doi: 10.1097/SLA.0000000000003256, PMID: 30817349

[20] Song GM Liu XL Bian W Wu J Deng YH Zhang H. Systematic review with network meta-analysis: comparative efficacy of different enteral immunonutrition formulas in patients underwent gastrectomy. Oncotarget. (2017) 8:23376–88. doi: 10.18632/oncotarget.15580, PMID: 28423579 PMC5410311

[21] Yang FA Chen YC Tiong C. Immunonutrition in patients with pancreatic cancer undergoing surgical intervention: a systematic review and Meta-analysis of randomized controlled trials. Nutrients. (2020) 12:2798. doi: 10.3390/nu12092798, PMID: 32932707 PMC7551679

[22] Li XK Zhou H Xu Y Cong ZZ Wu WJ Luo J. Enteral immunonutrition versus enteral nutrition for patients undergoing oesophagectomy: a systematic review and meta-analysis. Interact Cardiovasc Thorac Surg. (2020) 30:854–62. doi: 10.1093/icvts/ivaa022, PMID: 32206808

[23] Zhuo ZG Luo J Song HYDTN Alai GH Shen X Lin YD. Is immunonutrition superior to standard enteral nutrition in reducing postoperative complications in patients undergoing esophagectomy? A meta-analysis of randomized controlled trials. J BUON. (2021) 26:204–10. PMID: 33721453

[24] Kanekiyo S Takeda S Iida M Nishiyama M Kitahara M Shindo Y. Efficacy of perioperative immunonutrition in esophageal cancer patients undergoing esophagectomy. Nutrition. (2019) 59:96–102. doi: 10.1016/j.nut.2018.08.006, PMID: 30468936

[25] Li XK Cong ZZ Wu WJ Xu Y Zhou H Wang GM. Enteral immunonutrition versus enteral nutrition for patients undergoing esophagectomy: a randomized controlled trial. Ann Palliat Med. (2021) 10:1351–61. doi: 10.21037/apm-20-1399, PMID: 33222455

[26] Xu J Zhong Y Jing D Wu Z. Preoperative enteral immunonutrition improves postoperative outcome in patients with gastrointestinal cancer. World J Surg. (2006) 30:1284–9. doi: 10.1007/s00268-005-0756-8, PMID: 16830214

[27] Rey-Ferro M Castaño R Orozco O Serna A Moreno A. Nutritional and immunological evaluation of patients with gastric cancer before and after surgery. Nutrition. (1997) 13:878–81. doi: 10.1016/S0899-9007(97)00269-4, PMID: 9357024

[28] Costa GT Vasconcelos QDJS Aragão GF. Fructooligosaccharides on inflammation, immunomodulation, oxidative stress, and gut immune response: a systematic review. Nutr Rev. (2022) 80:709–22. doi: 10.1093/nutrit/nuab115, PMID: 34966938

[29] Lukaski HC Garcia-Almeida JM. Phase angle in applications of bioimpedance in health and disease. Rev Endocr Metab Disord. (2023) 24:367–70. doi: 10.1007/s11154-023-09799-0 36944817 PMC10030341

[30] Amano K Bruera E Hui D. Diagnostic and prognostic utility of phase angle in patients with cancer. Rev Endocr Metab Disord. (2023) 24:479–89. doi: 10.1007/s11154-022-09776-z, PMID: 36484944

[31] da Silva1 BR Orsso1 CE Gonzalez2 MC Sicchieri3 JMF Mialich3 MS Jordao3 AA. Phase angle and cellular health: inflammation and oxidative damage. Rev Endocr Metab Disord. (2023) 24:543–62. doi: 10.1007/s11154-022-09775-0, PMID: 36474107 PMC9735064

[32] Detopoulou P Fragopoulou E Nomikos T Antonopoulou S. Associations of phase angle with platelet-activating factor metabolism and related dietary factors in healthy volunteers. Front Nutr. (2023) 10:1237086. doi: 10.3389/fnut.2023.1237086, PMID: 38024339 PMC10655008

[33] Di Renzo L Marchetti M Cioccoloni G Gratteri S Capria G Romano L. Role of phase angle in the evaluation of effect of an immuno-enhanced formula in post-surgical cancer patients: a randomized clinical trial. Eur Rev Med Pharmacol Sci. (2019) 23:1322–34. doi: 10.26355/eurrev_201902_17027, PMID: 30779100

