# Supplementary material for: Efficacy of a new immunonutrition formula with extra virgin olive oil in the reduction of complications in surgeries of upper digestive tract tumors
Source: Front Nutr. 2024 May 28;11:1384145. doi: 10.3389/fnut.2024.1384145 (PMC11165349; doi:10.3389/fnut.2024.1384145)
Supplement: Supplementary file 1 [file Table_1.DOCX]

Supplementary Material

Article Title

Efficacy of a New Immunonutrition Formula with Extra Virgin Olive Oil in the Reduction of Complications in Surgeries of Upper Digestive Tract Tumours

**Rocío Villar Taibo, Alfonso Vidal-Casariego*, Alicia Santamaría Nieto, Ana Cantón Blanco, Ana B. Crujeiras, Gloria Lugo Rodríguez, Gemma Rodríguez Carnero, Francisco Pita Gutiérrez, Antía Fernández Pombo, Everardo Díaz López, Andrea Román Eyo, Uxía Rodríguez Lavandeira, Alberto Pena Dubra and Miguel Ángel Martínez-Olmos.**

*** Correspondence:**

Alfonso Vidal-Casariego

[alfonso.vidal.casariego@sergas.es](mailto:alfonso.vidal.casariego@sergas.es)

Tel.: +34 981176442

# Supplementary Data

**Technological details of the extraction, preparation, separation, processing of arginine and extra virgin olive oil.**

# L-arginine is produced through fermentation by bacteria culture in a medium with optimal conditions to produce and secrete it. Manufacturing consists of several processes: L-arginine synthesis, secretion, collection and purification. Mutants from the genus Corynebacterium and Bacillus or recombinant DNA Escherichia coli are generally used for L-arginine production. The mutations or recombinant genes carried by them confer these strains the property of having a strong biosynthetic pathway towards the production of this amino acid. Fermentation occurs in a fermentation tank where L-arginine producing bacteria strains, substrates and some other conditions are present. The milieu includes sources of carbon, nitrogen, minerals and proper quantities of nutrients that bacteria require for their optimal growing and L-arginine production. Carbon sources can be several carbohydrates, organic acids or alcohols. Ammonia or its salts are generally employed as nitrogen source. Potassium monophosphate, magnesium sulphate, sodium chloride or calcium chloride are among the most used minerals and thiamine and yeast extracts are the main vitamin sources. Culture needs aerobic conditions. For that reason, the culture is under stirring with aeration, at a temperature between 30-38oC, pH tends to be neutral, between 6.5-7.2 and can be adjusted with some acids, bases or other buffer solutions. In general, a culture from 2 to 5 days is enough to obtain a good concentration of L-arginine in liquid medium. After fermentation, microorganisms and proteins are removed by membrane filtration. The filtrate is charged to a resin tower in order to remove impurities, such as organic acids and other amino acids. Then, the product is concentrated to form L-arginine crystals, which are separated by centrifugation. These crystals are dissolved in pure water and ultrafiltered. As L-arginine dissolves better in hot water, this solution must be cooled to 10oC to optimise the obtention of pure L-arginine crystals. Finally, crystals are dried with a dryer to minimise moisture concentration.

# The olive reception takes place in the reception hopper, where prior to its unloading the correct state of it has been verified. Next, the olive goes to the cleaning process, where the leaves and stems than can accompany the fruit are removed. Then the washing takes place, where the olive is washed to remove possible dirt or dirt in it (optional for some lines depending on the dirt that the olive has) and in the last phase, a shower with water occurs drinking of the olive. The olive is then stored in the storage hoppers and from these phases in the blenders where it is subjected, for an approximate time of an hour and at a temperature of approximately 25ºC, to a milkshake that facilitates the coalescence of the oil, adding talcum powder if necessary in a proportion always less than 1%. At this point, the horizontal centrifugation phase is passed in which the oil phase (oil) is separated from the aqueous phase and the solid phase in horizontal centrifuges. The small amounts of water and solids that may accompany the oil are separated in the vertical centrifugation phase where the oil is cleaned by centrifugal force and injected water. The outlet oil of the vertical centrifuges is conducted to the decanting stage, in which the oil is stored in decanters installed in series for the complete separation of moisture and impurities from the oil. From here, the oil is taken to storage in the cellar, where the final storage of the oil takes place until it is packed or sold in bulk. For the packaged oils, previously, the filtering stage is carried out, which consists in the removal of moisture and impurities contained in the oil by passing it through tarps, with filtering materials (cellulose) that have a porosity that makes them very suitable as filter material). In the packaging there are two basic operations that take place simultaneously: the filling and dosing of containers. It must be done with machinery that guarantees precision and cleanliness. The rest of the phases can be carried out in a simple way: closed containers, labelling, embedded and palletized. For unpackaged oils, bulk shipping takes place, where the oil passes from the celler to a tank suitable for transporting food products by means of a transfer pump.
