# Supplementary material for: Efficacy of a new immunonutrition formula with extra virgin olive oil in the reduction of complications in surgeries of upper digestive tract tumors
Source: Front Nutr. 2024 May 28;11:1384145. doi: 10.3389/fnut.2024.1384145 (PMC11165349; doi:10.3389/fnut.2024.1384145)

METHODS

- Clinical trial
- Randomized
- Double-blind
- Parallel
- Multicentre

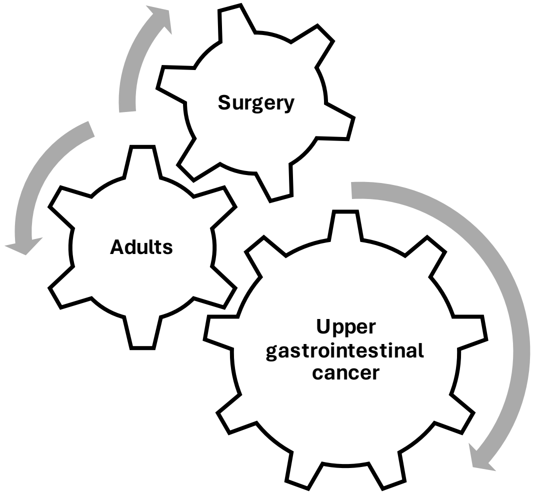

FORMULAS (isocaloric & isoproteic)

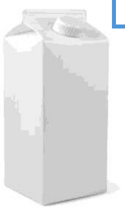

IMMUNONUTRITION-ONS

- ✓ 600 kcal
- ✓ 32 g proteins
- ✓ **8 g arginine**
- ✓ 3,000 mg **EPA & DHA**
- ✓ 800 mg nucleotides
- ✓ 9.5 g EVOO
- ✓ 8.2 g fibre (100/0)

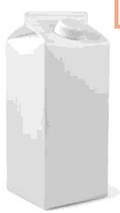

CONTROL-ONS

- ✓ 636 kcal
- ✓ 31.5 g proteins
- ✓ 1 g arginine
- ✓ Without EPA & DHA
- ✓ Without nucleotides
- ✓ Without EVOO
- ✓ 0 g fibre

Nutritional composition of the formulas under study per 400 ml.

5 days

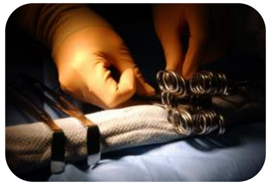

Surgery complications

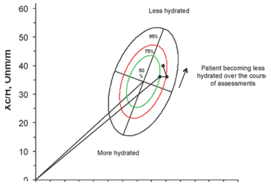

Body composition

RESULTS

IMMUNONUTRITION-ONS

↓ **REINTERVENTION RATE**

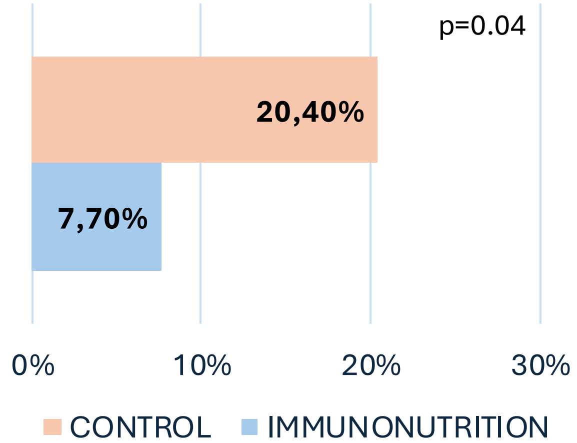

IMMUNONUTRITION-ONS

↑ **CELULAR FUNCTION**

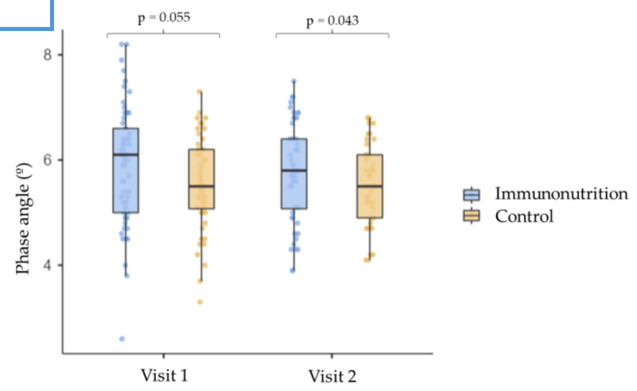

Supplement: Supplementary file 2 [file Image_1.pdf]
